# Guttural pouch tympany or empyema in 38 ٍStraight Egyptian Arabian foals: clinical presentation, diagnostic features and outcome

**DOI:** 10.1186/s12917-026-05810-3

**Published:** 2026-08-19

**Authors:** Ashraf M. Abu-Seida, Hanaa A.E. Asfour, Mohammad A.H. Abdulredha, Mohamed K. Derbala

**Affiliations:** 1 Department of Surgery, Anesthesiology and Radiology, Faculty of Veterinary Medicine, Cairo University, PO: 12211, Giza Square, Giza, Egypt; 2 Mastitis and Neonatal Diseases Department, Animal Reproduction Research Institute (ARRI), Agriculture Research Center (ARC), Giza, Egypt; 3 Ministry of Defense of Kuwait, Military Sports Federation, Sabhan City, Kuwait; 4 Diagnostic Imaging and Endoscopy Unit - Animal Reproduction Research Institute (ARRI), Agriculture Research Center (ARC), PO: 12556, 5 - Hadyek EL-Behoth St. Haram, Giza, Egypt

**Keywords:** Chondroids, Endoscopy, Guttural pouch empyema, Guttural pouch tympany, Laser

## Abstract

**Background:**

As a breed, Straight Egyptian Arabian horses are susceptible to many disorders that mostly affect the head, such as guttural pouch (GP) tympany and empyema. This retrospective study evaluated the findings of clinical examination, ultrasonography, endoscopy, laboratory analyses and outcome of treatment of 38 Straight Egyptian Arabian foals with GP tympany and empyema.

**Methods:**

Data from medical records of foals diagnosed with GP tympany or empyema over a 2-year period (2024–2025) were reviewed. Foals with verified GP tympany or empyema who had complete medical records and had not previously received antibiotics and/or corticosteroids met the inclusion criteria. Foals with incomplete records, unconfirmed diagnosis or concurrent diseases likely to influence the evaluated variables were excluded. Outcomes of treatment (recovery and recurrence) were compared with diagnostic features and treatment modalities. The data were statistically analyzed.

**Results:**

Out of 237 foals presented with various disorders, 38 foals had GP tympany and empyema, representing 16.03% of the total examined foals. Out of 38 foals with GP tympany and empyema, there were 29 females (77.8%) and 9 males (22.2%). The frequencies of GP tympany and empyema were significantly higher in the first month of life (47.4%) than the 8-9-month period (10.5% ((*P* < 0.05). Bilateral GP tympany and empyema were recorded in 30 foals (78.9%). While eight cases (21.1%) had unilateral GP tympany and/or empyema. The studied foals had GP tympany (25/38, 65.8%) or GP empyema (13/38, 34.2%). Out of 25 foals with GP tympany, five foals (20%) had complicated GP tympany with microbial infections. Ultrasonography examination of the GP tympany and empyema had a diagnostic value in 15.8% of the cases. In foals with GP tympany, endoscopy revealed distention of the afflicted GP with air, dorsal or lateral nasopharyngeal compression, and a stretched, thinning, pale mucosal lining. However, mucopurulent material and hyperemic, uneven GP mucosa were observed in one or both GPs in foals with GP empyema. Foals with uncomplicated GP tympany had normal hematology and serum chemistry. While foals with GP empyema and complicated GP tympany exhibited mild to moderate leukocytosis, neutrophilia with left shift in severe infection, hyperfibrinogenemia, mild hyperproteinemia, mild hyperglobulinemia (in chronic cases), mild hypoalbuminemia (in chronic cases), and increased serum amyloid A. Out of 38 foals with GP tympany and empyema, 18 foals (47.4%) had microbial infections. The microbial isolates were double mixed infections (50%) and triple mixed infection (50%). The most commonly isolated bacteria were *α-hemolytic S. equi* ssp. *equi* (13/18, 72.2%). Out of 36 treated foals, 33 foals (91.7%) with GP tympany and empyema recovered after 4–6 weeks of treatment by placement of a transnasal Foley catheter under the guidance of endoscopy with repeated lavage and administration of appropriate antibiotics. Three recurrent cases were successfully treated with transendoscopic laser fenestration of both GPs into pharyngeal cavity.

**Conclusions:**

Arabian foals with GP tympany and/or empyema have characteristic clinical signs and endoscopic findings and nonspecific findings of hematology and serum chemistry. Although ultrasonography is a helpful diagnostic technique for foals with GP empyema and complicated GP tympany, endoscopy remains the gold standard for confirming the diagnosis of GP illness. All foals with GP empyema and complicated tympany should undergo culture and sensitivity test for a proper diagnosis and course of therapy because all cases of GP empyema and complicated cases of GP tympany have double and triple mixed microbial infections.

## Background

Among domesticated animals, horses and only a few other species have a distinctive feature called the guttural pouch (GP). GP is an air-filled out pouching or diverticulum of the auditory tube (Eustachian tube), with an average volume of 300–500 mL. The auditory tube travels from the middle ear’s tympanic chamber to the nasopharynx. It is located in the area of the head ventral to the base of the skull and the atlas wing, dorsal to the nasopharynx and larynx, and the mandibular ramus is the caudal border [1].

The median septum, a small membrane where the medial walls of the left and right GPs are next to one another, divides them on the midline [2]. GPs balance pressure throughout the eardrum by serving as air reservoirs and being connected to the middle ear through the Eustachian tubes. Moreover, GPs warm breathed air, serve as a vocal resonator, cool down the brain by cooling the internal carotid arteries that supply the brain, and control intracranial blood pressure, especially when exercising [1, 2].

Serious bacterial and fungal infections can occur in the GPs. These infections can induce several clinical signs such as swelling of the GP, dysphagia, facial paralysis, or potentially fatal bleeding. Tympany, empyema, mycosis, rupture of the ventral straight muscles, foreign bodies, neoplasia, and temporohyoid osteoarthropathy are among the GP disorders that have been reported [3–5].

When one or both GPs are swollen with air, the condition is known as GP tympany. Neonatal foals with congenital GP tympany have swollen cheeks. Although the unilateral variant is more prevalent, it can be bilateral or unilateral [6]. The mucosal flap (plica salphingopharyngeus) covering the GP opening in afflicted foals is thought to function as a one-way valve, allowing air to enter but not to depart. Although the exact cause is uncertain, suggestions include pharyngeal muscle weakness or mucosal fold dysfunction [7]. Females are more likely than males to have GP tympany at a ratio of 2.92:1, and the Paint horse and Arabian breeds may be overrepresented [8]. The estimated heritability is 0.81 ± 0.16 [9], with a mixed monogenic-polygenic mode of heredity [10] and a polygenic form of inheritance [9].

A fungal infection of one or both GPs is known as guttural pouch mycosis. Most frequently, fungal plaques develop inside the GPs along the walls of the main blood vessels, including the maxillary, internal, and external carotid arteries [11]. *Aspergillus* is the most often found species of fungus, although the exact origin of GP mycosis is uncertain [12, 13]. Moderate to severe nosebleed and dysphagia are the most typical clinical symptoms. Horner’s syndrome, white nasal discharge, aberrant head posture, throatlatch pain, and irregular breathing sounds are other uncommon symptoms. The gold standard for diagnosing GP mycosis is endoscopy of the GP. Topical antifungal medicines, either with or without systemic antifungal medications, are infused into the afflicted GP as part of medical treatment for GP mycosis [14]. GP mycosis can be treated surgically using balloon catheter blockage of the internal carotid artery and transarterial coil or plug embolization. In order to prevent bleeding, the majority of treatments try to block the damaged artery [15–18].

The clinical signs of GP empyema include cough, pyrexia, nasal discharge, epistaxis, difficulty swallowing, facial drooping, head tilt, roaring (noisy breathing), and lymph node abscessation. It is caused mainly by a local infection with *Streptococcus equi* ssp. *equi* [8, 19]. “Chondroids” are the result of formation of hard, inspissated pus in chronic cases of GP empyema. The diagnosis is based upon the clinical signs, endoscopy, ultrasonography and microbiological examinations [20, 21]. Prior studies have looked at medical treatment and reported efficacy with topical antibiotic therapy or lavage with warm water until pus is not produced any more [20, 21]. Therefore, the treatment options include flushing, antibiotics or surgery. Both endoscopic transnasal Foley catheter insertion into the GP pharyngeal aperture and transpharyngeal diode laser fenestration are used to treat GP tympany and chronic GP infection [19]. About 80% of Foley catheter placements were successful [19]. The prognosis of GP empyema varies from good for minor infections to guarded for mixed and severe infections [21].

This retrospective study evaluated the results of clinical examination, ultrasonography, endoscopy, and laboratory findings in 38 Straight Egyptian Arabian foals with GP tympany and empyema and their treatment outcomes.

## Animals, materials and methods

### Animals

In the present study, all international and institutional regulations of the animals^,^ studies were followed. Ethical approval was obtained from the Institutional Review Board (IRB) at Animal Reproduction Research Institute (ARRI), Agriculture Research Center (ARC), Giza, Egypt (Approval number: 129241). During the period between January 1, 2024 and December 31, 2025, 237 Straight Egyptian Arabian foals with various disorders were admitted to Derbala Equine Clinic, Giza, Egypt. Out of these foals, 38 animals had confirmed GP tympany or empyema. As a routine in the clinic, an informed consent was obtained from all owners of these 38 foals to participate research (sample collection and treatment). The diagnosis of GP tympany and empyema was based on the results of clinical examination, ultrasonography, endoscopy and laboratory analyses.

Foals were included if they met all of the following criteria:

- Age ≤ 9 months at the time of presentation.
- Had confirmed GP tympany and/or empyema based on the clinical signs, ultrasonography, endoscopy, and laboratory findings.
- Availability of all complete clinical reports (signalment, history, findings of clinical, ultrasonographic and endoscopic examinations, blood work findings, microbiological culture and antimicrobial sensitivity results, treatment and outcomes).
- Not previously received antibiotics and/or corticosteroids.

The exclusion criteria included; foals with incomplete medical records, had unconfirmed diagnosis of GP disease, had undergone previous GP surgery, had concurrent severe systemic disorders such as severe pneumonia and septicemia as well as had contaminated microbiological samples.

### Clinical examination

The foals underwent a thorough clinical examination, including body temperature, respiratory and heart rates, mucous membranes and lymph nodes. The general health condition was assessed by determining demeanour, appearance of hair coat, skin elasticity, and position of the eyes in relation to the sockets. The parotid regions were carefully examined.

### Ultrasonography examination

Ultrasonography of the GP tympany and empyema was carried out using ultrasound device (Sonoscape E1v, SonoScape Medical Corp., Shenzhen, Guangdong, China) supplied with 4 MHz linear and convex probes for scanning of the parotid region after application of ultrasound coupling gel. All ultrasonography findings were reported.

### Endoscopic examination

Fiberoptic endoscopy was used for diagnosis and treatment of the foals (Olympus, CV-240, optical CO. LTD, Japan). To ensure easy manipulation and comfort of the foals, they were placed in stocks and sedated with 500 µg/kg BW intravenous Xylazine HCl (Xylaject 2%^®^, ADWIA, Egypt) and 30 µg/kg BW Butorphanol (Morphasol^®^, Livisto, Barcelona, Spain) [22].

Before each examination, the endoscope was cleaned and disinfected according to the manufacturer’s recommendations. A sterile Foley catheter was introduced under endoscopic guidance into the affected GP. Just prior to entering the GP opening, the catheter’s sterile, hygienic plastic sheath was opened by applying pressure to the sheath’s end using a stylet. In foals with GP empyema and complicated GP tympany, the purulent material was aspirated directly from the GP using sterile technique while minimizing contact with the upper respiratory mucosa for microbiological analyses. In cases of uncomplicated GP tympany, the microbiological samples were collected from the effluent of lavage of the GPs. The catheter and collection materials were sterile and used for a single animal only.

**Fig. 1:**
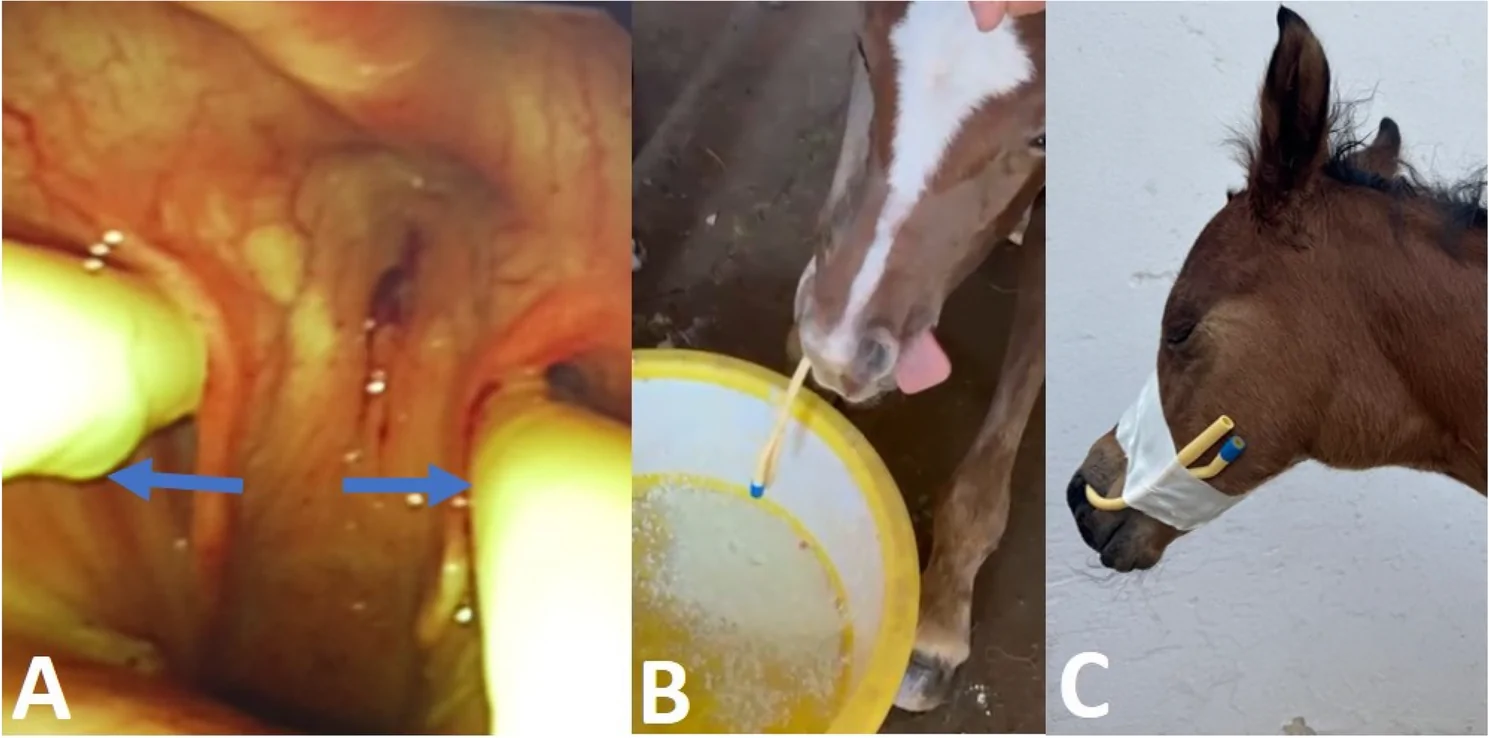
**A** Endoscopy image showing inserting of two Foley catheters into the guttural pouches, (**B**) Evacuation of pus in a two-month-old Straight Egyptian Arabian foal and (**C**) Fixation of the Foley catheter to the foal’s head

In order to see the pharyngeal opening of the right GP, the endoscope was first placed into the left nasal opening and then moved into the ventral nasal meatus until it reached the pharynx. A curved sterile stylet was used to help placement of a new sterile 24 French Foley catheter (Foley Balloon catheter^®^ Sunbow Medical Instruments Co., Ltd, Nigbo, China) via the right pharyngeal opening into the GP. The stylet was removed from outside the right nostril after the Foley catheter was inserted into the right GP and inflated with 20 mL of sterile normal saline solution. Similarly, whenever the other GP was affected, it was catheterized **(**Fig. 1A**)**. After catheterization of GPs, the pus was evacuated **(**Fig. 1B**)** and the catheter was fixed to the animal’s head as shown in Fig. 1C.

### Laboratory analyses

#### Hematological and serum biochemical analyses

The following blood samples were collected from the jugular vein of all foals: 5 mL of EDTA blood for hematological examination and 10 mL of whole blood for serum biochemical analyses. The measured variables included the packed cell volume, red blood cell count, total leukocyte count, blood platelet count, concentrations of fibrinogen, total protein, albumin, globulin, and serum amyloid A (SAA) using automated blood analyzer and automated clinical chemistry analyzers (Abbott Diagnostics Division, Baar, California, USA). A differential leukocyte count was performed in foals with leukocytosis (> 12,500 leukocytes/µL blood). Results were compared to reference intervals previously reported [23].

#### Microbiological examination

After catheterization of GPs, the samples were taken into sterile falcon tubes and sent to the microbiology lab for culture, isolation and identification of microorganism and antibiotics sensitivity test as well. All microbiological isolations were performed according to De Vos et al. [24]. A loopful of each incubated sample was cultivated on blood agar (with 5% defibrinated sheep blood), Edward’s media, Eosin-methylene blue agar (EMB), MacConkey agar, Mannitol salt agar, Sabouraud dextrose agar (SDA) containing 0.05 mg/mL chloramphenicol, and HiCrome Candida Differential Agar (HiMedia Laboratories, Mumbai, India). After being incubated at 37 °C for 24 to 48 h, the bacterial growth on the different agar plates was quantified and identified. After additional 24 h of incubation under the same conditions, no discernible growth plates were reexamined [25]. Hemolytic behavior and morphological characteristics were used to identify colonies; suspicious colonies were gathered and examined under a microscope on Gram-stained films before being moved to semisolid agar for additional biochemical identification of each microbe [26]. A GP infection was indicated by the emergence of two or more colonies of any species. Plates of Sabouraud dextrose agar and Candida differential agar were incubated at 27 and 37^◦^C, respectively. After 24 h, the development of yeast and fungi was measured and identified. Plates that showed no growth were incubated at 27^◦^C for an additional four days [26, 27]. Then, the plates were incubated at room temperature (20–25 °C) up to 3–4 weeks to avoid dryness of the agar. The results were recorded as negative for fungal growth after 4 weeks of follow up the plates. The sensitivity tests were conducted according to the guidelines of the Clinical & Laboratory Standards Institute [28].

### Treatment

In case of GP empyema, the affected pouch was flushed every two days with one-liter of sterile warm normal saline solution (200 mL for five successive times) for 4–6 weeks. Then, the suitable antibiotics, according to the results of the sensitivity test, were given intramuscularly for five days and topically inside the GP via the catheter for also five days. Stall rest and isolation of the affected foal were carried out for three weeks in order to give the respiratory epithelium time to heal and to avoid spreading of infection. Dust was kept to a minimum and feeding was done on the ground to allow for drainage. While in case of GP tympany, the affected pouch was flushed every three days with sterile warm normal saline solution for 4–6 weeks as mentioned before.

**Fig. 2:**
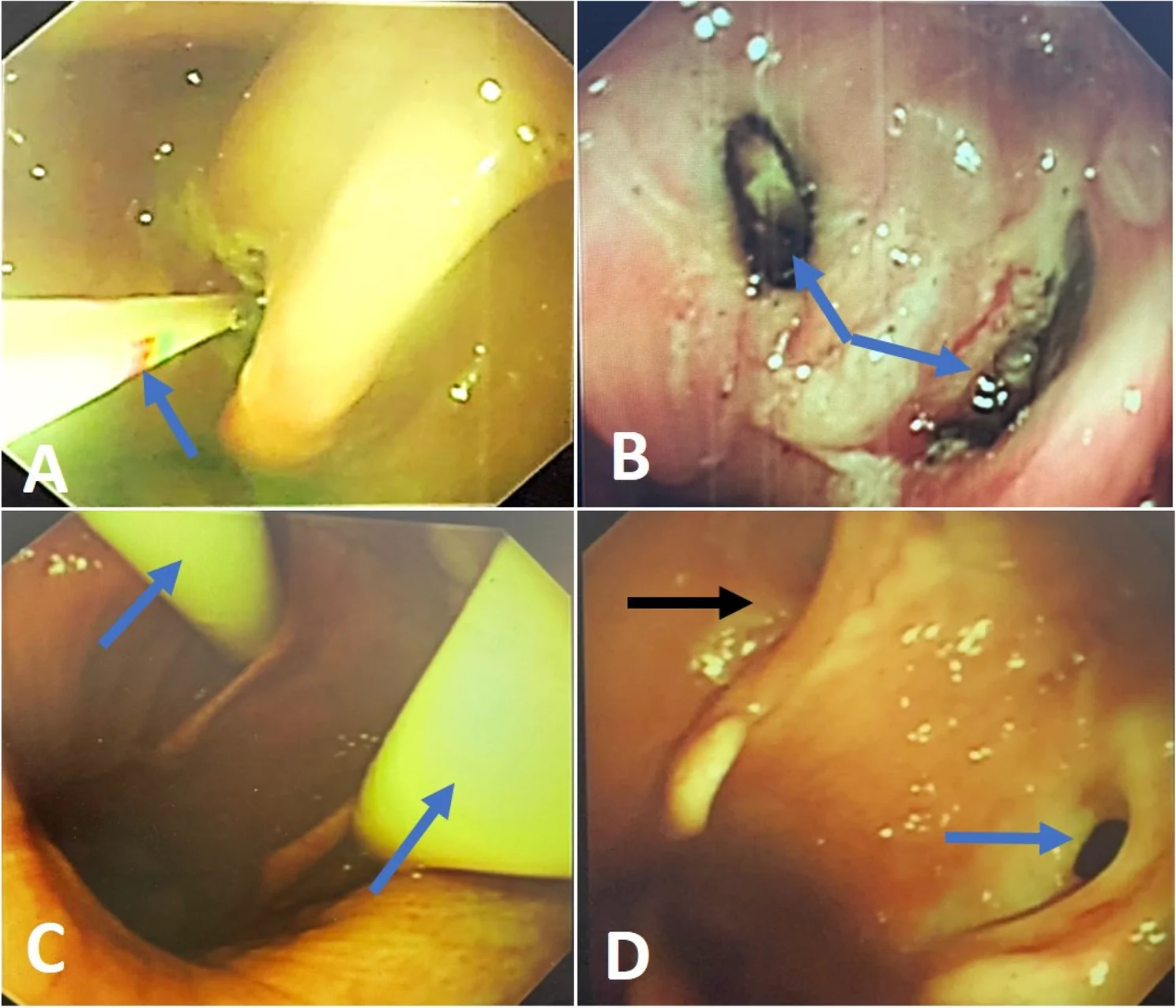
Endoscopic images showing (**A**) Diode laser during fenestration of guttural pouch (Blue arrow), (**B**) Two induced openings (Blue arrows) in the two guttural pouches after fenestration with Diode laser in a foal with bilateral guttural pouch tympany, (**C**) Fixation of two Foley catheters (Blue arrows) into both guttural pouches in a foal with bilateral guttural pouch tympany and (**D**) The created patent opening after removal of the Foley catheter (Blue arrow) and the natural opening (Black arrow)

In recurrent cases, laser fenestration was conducted. A hole was made close to the pharyngeal opening of the affected GP using the MLT-Diode Laser Premium (MLT-Medizinische Laser Technologie GmbH, Germany) with a wavelength of 980 nm and an adjusted power of up to 18 watts (W) **(**Fig. 2**)**. Following the introduction of the endoscopy through the nostril and the imaging of the GP and pharyngeal region, a laser filament was inserted through the endoscopy’s working channel until its working end attached to the inflated GP. A hole was then made close to the natural GP orifice, and the Foley catheter was inserted through the operated opening and fixed by inflation with 20 mL of normal saline. Outcomes of treatment (recovery and recurrence) were compared with diagnostic features and treatment modalities.

### Statistical analysis

The program IBM SPSS Statistics 22.0 was applied for assessment. Frequencies were determined for each clinical and laboratory variable. The Wilk-Shapiro test was carried out to evaluate normality of data. Means ± standard deviations were calculated for normal data and medians for non-normal data. Differences in occurrence at various ages were analyzed using a one-way analysis of variance and the post hoc Bonferroni test. A value of *P* < 0.05 was considered significant.

## Results

### Clinical findings

Over the two-year-study period, foals with GP tympany and empyema constituted 16.03% of the foal patients treated for various disorders. The length of illness ranged from one to ten days (median, four days).

Out of 38 foals with GP tympany and empyema, 29 were females (77.8%) and 9 were males (22.2%). The diseased foals ranged in age from one week to 9 months (median, 2.1 months) with 47.4% of the foals being less than one month of age. The incidence of GP tympany and empyema was significantly higher (*P* < 0.05) in the first month of life (47.4%) than in the 8-9-month period (10.5%) as shown in Table 1.

**Table 1:** Distribution of guttural pouch tympany and/or empyema among the age, gender and laterality of the examined Arabian foals

Items		Number	%
Age	One week - One month	18	47.4
	2–7 months	16	42.1
	8–9 months	4	10.5
Gender	Female	29	77.8
	Male	9	22.2
Laterality	Bilateral	30	78.9%
	Unilateral	8	21.1%

**Fig. 3:**
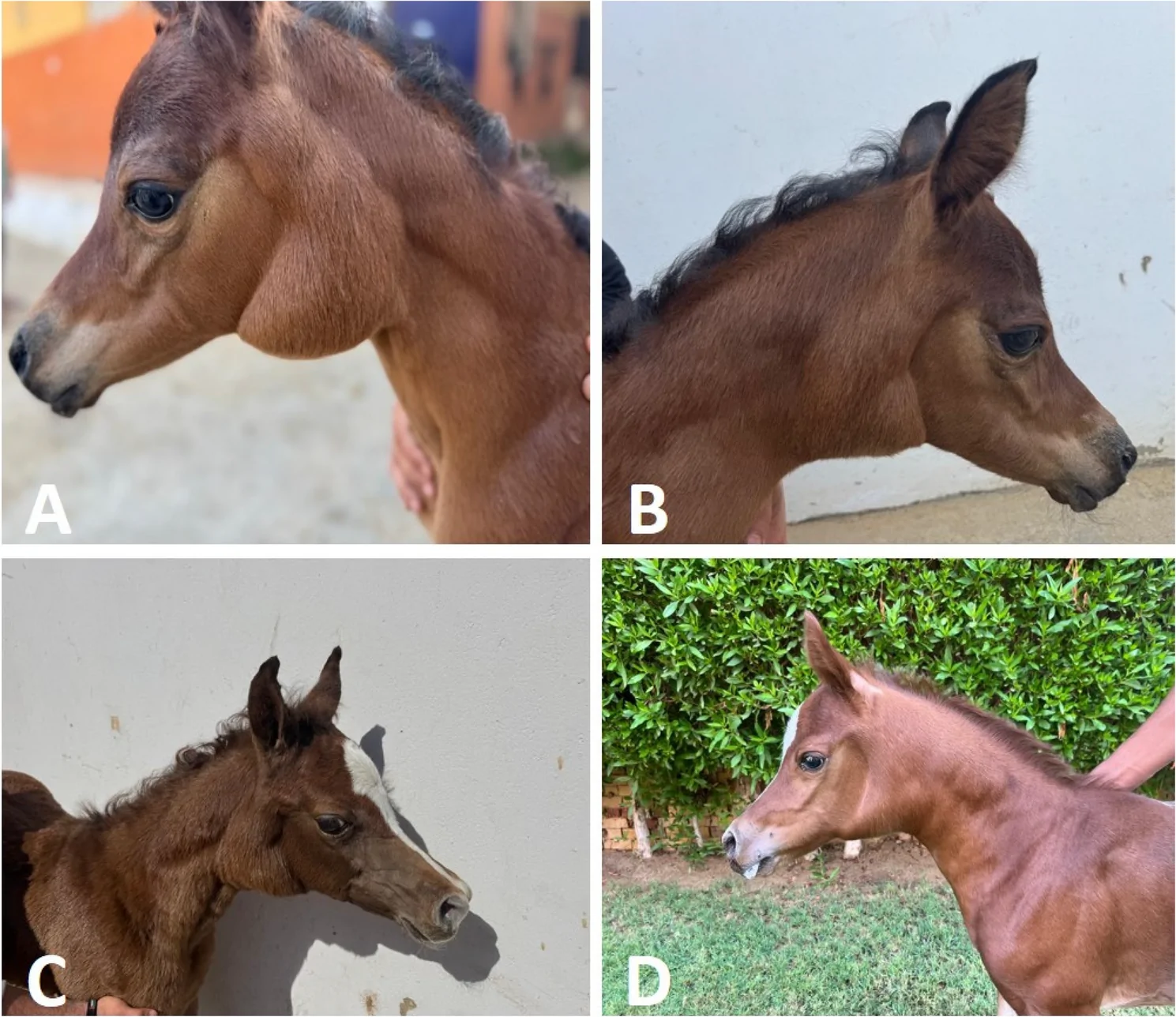
**A**&**B** Bilateral guttural pouch tympany in a three-week-old Straight Egyptian Arbian filly. **C** Right guttural pouch empyema in a two-month-old Straight Egyptian Arbian foal. **D** Left guttural pouch tympany in a three-month-old Straight Egyptian Arbian foal

Bilateral GP tympany and empyema were recorded in 30 foals (78.9%) as shown in Figs. 3A&B. While eight cases (21.1%) had unilateral GP tympany or empyema (Figs. 3C&D). The recorded cases included; GP tympany (25/38, 65.8%) and GP empyema (13/38, 34.2%). Out of 25 foals with GP tympany, five foals (20%) had complicated GP tympany with microbial infections.

Clinically, foals with uncomplicated GP tympany (*n* = 20) had normal to mildly reduced body condition and normal body temperature (37.4–38.5 °C, 37.8 ± 0.4 °C), heart rate (62–90 beats/min, median: 76 beats/min), respiratory rate (19–38 breaths/min, median: 29 breaths/min), and pink moist mucous membrane with capillary refill time < 2 s. While foals with complicated GP tympany (*n* = 5) and GP empyema (*n* = 13) exhibited moderate to poor body condition, high body temperature (38.9–40.6 °C, 39.6 ± 0.5 °C), tachycardia (85–135 beats/min, median: 102 beats/min), tachypnea (30–62 breaths/min, median: 48 breaths/min), and pink to congested mucous membranes with capillary refill time 2–3 s. The retropharyngeal and intermandibular lymph nodes were enlarged in 3/25 cases (12%) of GP tympany and 9/13 cases (69.2%) of GP empyema.

Foals with GP tympany represented 25/38 (65.8%) and exhibited a tympanic swelling at the parotid region. Regarding gender, 19/25 cases (76%) with GP tympany were females and 6/25 cases (24%) were males. Most of these cases (15/25, 60%) were reported in the first month of age (Figs. 3A&B). Out of 25 cases of GP tympany, bilateral GP tympany was recorded in 20 foals (80%). While 5 cases (20%) were unilateral.

**Fig. 4:**
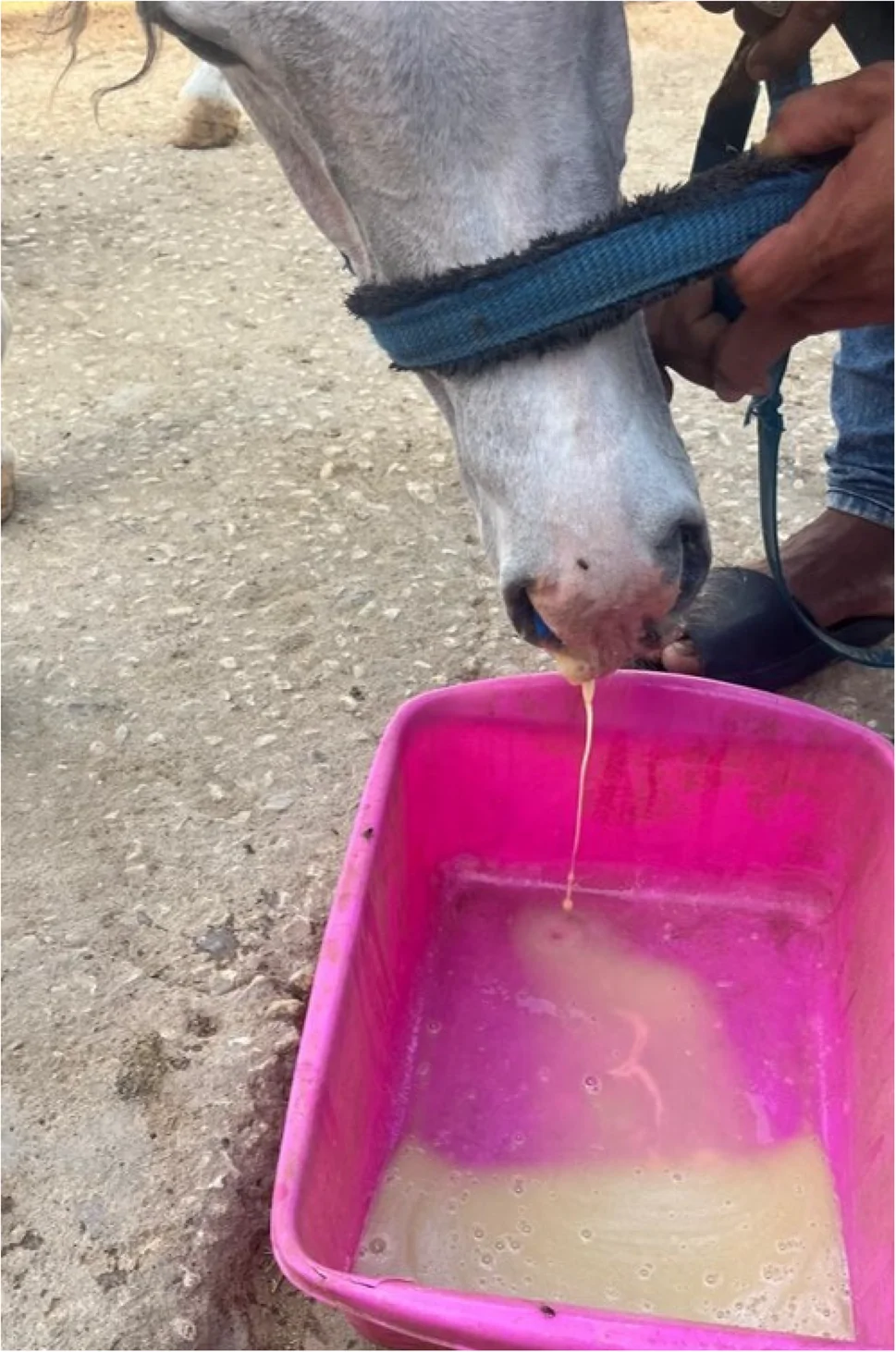
A five-month-old filly with right guttural pouch empyema showing unilateral purulent nasal discharge

**Fig. 5:**
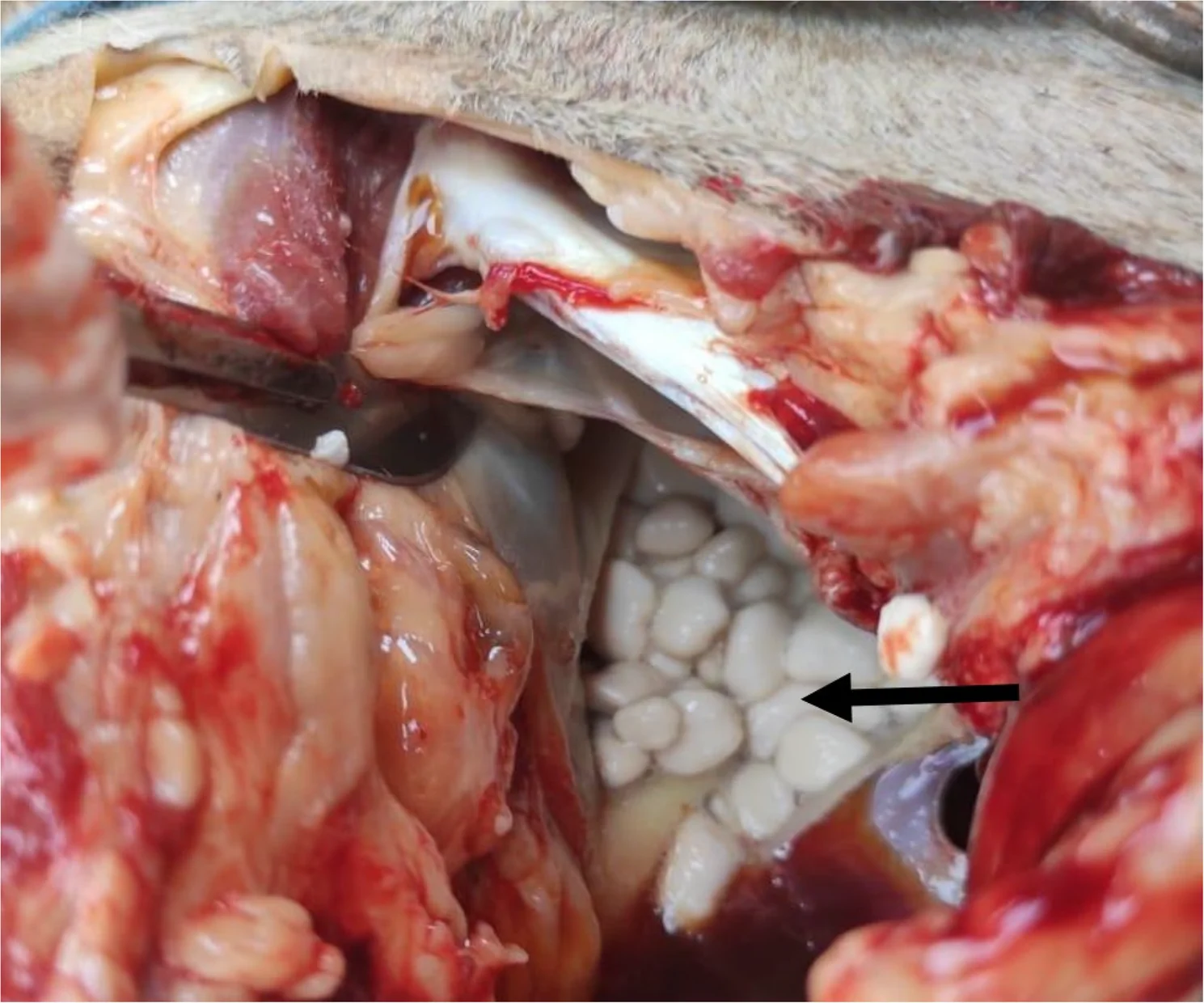
Post-mortem image of a six-month-old foal with guttural pouch empyema showing chondroids (Black arrow)

Foals with GP empyema represented 13/38 (34.2%). Regarding gender, 10/13 cases (76.9%) were females and 3/13 cases (23.1%) were males. Out of 13 cases of GP empyema, 10 foals (10/13, 76.9%) had bilateral GP empyema. While 3/13 cases (23.1%) were unilateral. The affected foals had common clinical signs such as cough, fever, purulent nasal discharge (Fig. 4), epistaxis, difficulty swallowing, facial drooping, head tilt, roaring with lymph node abscessation. Milk occasionally emerged from the nose. In one chronic case, hard casiated pus was formed “chondroids” as shown in Fig. 5. This case died before treatment due to metastatic strangles. Two complicated cases of GP empyema showed mixed bacterial and fungal infections (2/13, 15.4%). One of them suffered from bilateral epistaxis and neurological symptoms (head shaking and tremors) and died after 12 h of admission, before treatment. She had double infection with α-hemolytic *Streptococcus equi* subspecies *equi* and *Aspergillus niger*. The second case had mixed infection with three pathogens, including α-hemolytic *Streptococcus equi* subspecies *equi*,* Escherichia coli* and *Candida albicans* as shown in Table 2.

**Table 2:** Types and frequency of microorganisms isolated from GP empyema (*n* = 13) and complicated GP tympany (*n* = 5) in the examined Stright Egyptian Arabian foals

Type of infection	Number of cases/ Percentage	
	No.	%
Mixed infections involving two pathogens (9 cases/ 50%)		
* α-hemolytic S. equi ssp. equi + Klebsiella pneumoniae*	3	16.70%
* β-hemolytic S. equi ssp. equi + Staphylococcus aureus*	1	5.55%
* α-hemolytic S. equi ssp. equi + E. coli*	1	5.55%
* α-hemolytic S. equi ssp. equi + Pseudomonas aeruginosa*	1	5.55%
* α-hemolytic S. equi ssp. equi + Aspergillus niger*	1	5.55%
* S. aureus + E. coli*	1	5.55%
* S. aureus + K. pneumoniae*	1	5.55%
Mixed infections involving three pathogens (9 cases/ 50%)		
* β-hemolytic S. equi ssp. equi + S. aureus + P. aeruginosa*	2	11.1%
* α-hemolytic S. equi ssp. equi + S. aureus + K. pneumoniae*	2	11.1%
* α-hemolytic S. equi ssp. equi + CNS + E. coli*	2	11.1%
* α-hemolytic S. equi ssp. equi + K. pneumonia + P. aeruginosa*	2	11.1%
* α-hemolytic S. equi ssp. equi + E. coli + Candida albicans*	1	5.6%
Total	**18**	**100%**

*α-*hemolytic *S. equi: α-*hemolytic *Streptococcus equi* subspecies *equi.* β-hemolytic *S. equi*: β-hemolytic *Streptococcus equi. K. pneumoniae: Klebsiella pneumoniae. S. aureus: Staphylococcus aureus. P. aeruginosa: Pseudomonas aeruginosa. E. coli: Escherichia coli*

*CNS* Coagulase negative staphylococci

### Ultrasonography findings

**Fig. 6:**
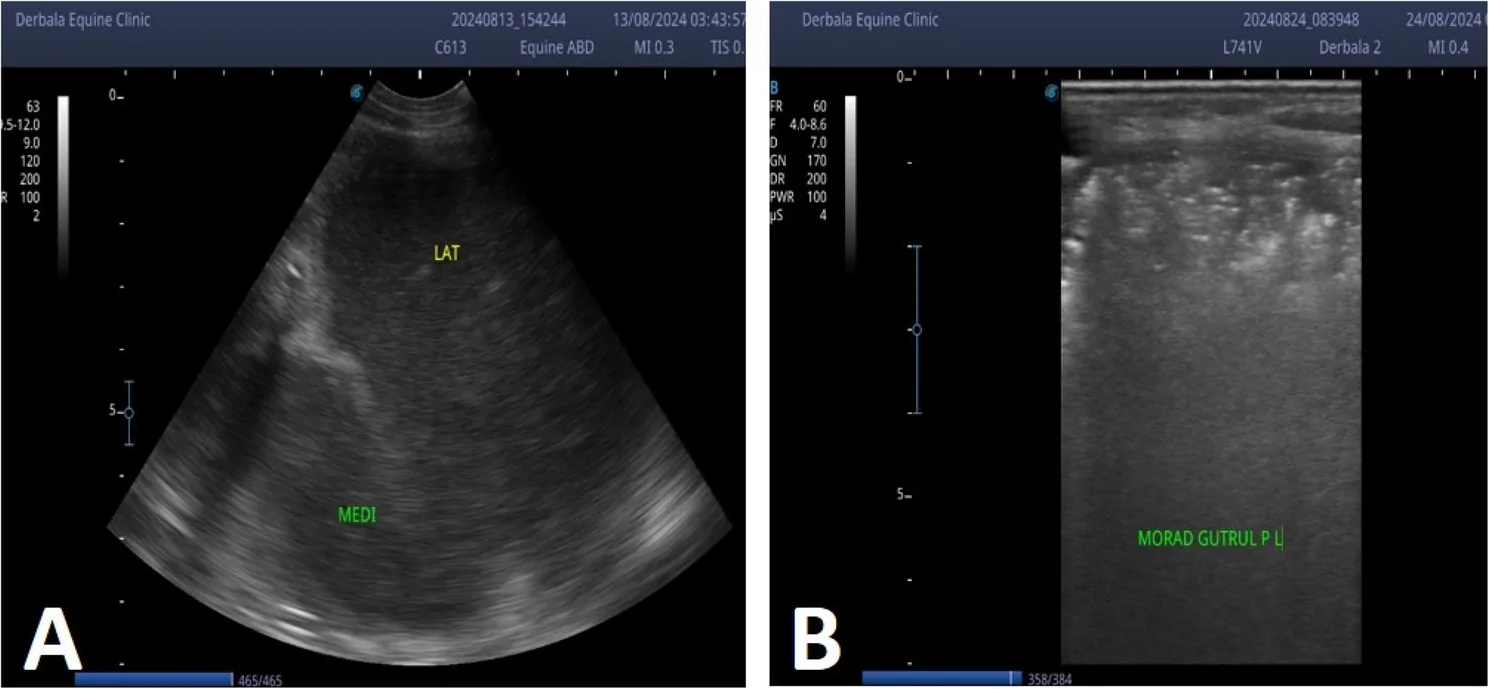
**A** Ultrasonography image of a foal with guttural pouch empyema showing hypoechoic homogenous fluid. **B** Ultrasonography image of a foal with guttural pouch empyema showing hypoechoic fluid intermingled with hyperechoic particles

Ultrasonography examination of the GP showed no diagnostic value except for 6/38 cases (15.8%). Four cases with GP empyema showed swollen GP with hypoechoic homogenous fluid (Fig. 6A). The other two foals showed swollen GP with hypoechoic fluid intermingled with hyperechoic dots (bacterial and fungal infections) as shown in Fig. 6B. The retropharyngeal lymph nodes seemed swollen and rounded on ultrasound scanning, losing their internal architecture. Foals with complicated GP tympany and GP empyema had hypoechoic to mixed echogenic lymph nodes. Different anechoic fluid or hyperechoic pockets indicating purulent debris or gas were observed in cases of lymph node abscessation.

### Endoscopic findings

Foals with GP tympany displayed acute distention of the afflicted GP with air that looked like an inflated, translucent balloon, dorsal or lateral nasopharyngeal compression, and a stretched, thinning, pale mucosal lining, and failure of the pharyngeal ostium to open normally during swallowing. In foals with complicated GP tympany, persistent pharyngeal compression, purulent material within the distended GP, and inflamed mucosa were seen.

Free-flowing, thick, creamy, or yellow-white mucopurulent material was observed in one or both GPs in foals with GP empyema. In addition, thick, hyperemic, uneven GP mucosa was visualized. In chronic cases of GP empyema, hard, inspissated pus nodules (chondroids) were seen.

### Laboratory findings

#### Hematologic and serum biochemical findings

Results of blood work showed normal hematology and serum chemistry in foals with uncomplicated GP tympany. While foals with GP empyema and complicated GP tympany (*n* = 18) exhibited mild to moderate leukocytosis (13000–20000 leukocytes/µL blood, median: 15400 leukocytes/µL, normal: 5000–12500 leukocytes/µL), neutrophilia with left shift in severe infection (9700–10200 cells/µL, median: 9900 cells/µL, normal: 2400–9500 cells/µL), hyperfibrinogenemia (450 ± 39 mg/dL, normal:100–400 mg/dL), mild hyperproteinemia (8.3–8.9 g/dL, median: 8.5 g/dL, normal: 6.0–8.0 g/dL), mild hyperglobulinemia in chronic cases (4.5–4.8 g/dL, median: 4.6 g/dL, normal:1.5–4.3 g/dL), mild hypoalbuminemia in chronic cases (2.1–2.4 g/dL, median: 2.3 g/dL, normal: 2.5–3.5 g/dL), and increased serum amyloid A (120–139 µg/mL, median: 128 µg/mL, normal: 20–100 µg/mL).

#### Microbiological findings

The results of microbial isolation are shown in Table 2. Out of 38 foals with GP tympany and empyema, 18 (47.4%) had microbial infections. The infected cases included all cases of GP empyema (*n* = 13) and 5/25 (20%) of foals with complicated GP tympany. The microbial isolates were double (50%) and triple (50%) mixed microbial infections. No single infection was reported in this study. The most commonly isolated bacteria were α-hemolytic *Streptococcus equi* subspecies *equi* (13/18, 72.2%), followed by *Klebsiella pneumoniae* (8/18, 44.4%) and *Staphylococcus aureus* (7/18, 38.9%) as shown in Table 2. The isolated fungi included *Aspergillus niger* (one case/18, 5.6%) and *Candida albicans* (one case/18, 5.6%). Results of sensitivity to antibiotics are illustrated in Table 3. The isolated bacteria from the foals with complicated GP tympany and empyema were highly sensitive (92.3–100%) to Imipenem, Meropenem, Levofloxacin, and Ciprofloxacin. While these bacteria were highly resistant (33.3–100%) to Penicillin, Lincomycin, and Kanamycin as shown in Table 3.

**Table 3:** Antibiotics susceptibility of the isolated bacteria from the examined foals

Antibiotics	Different bacterial isolates													
	α-hemolytic *S. equi* (13)		β-hemolytic *S. equi* (3)		*K.* pneumoniae (8)		*S.* aureus (7)		*P*. aeruginosa (5)		*E.* coli (5)		CNS (2)	
	S	R	S	R	S	R	S	R	S	R	S	R	S	R
	no (%)	no (%)	no (%)	no (%)	no (%)	no (%)	no (%)	no (%)	no (%)	no (%)	no (%)	no (%)	no (%)	no (%)
IPM	12 (92.3%)	1 (7.7%)	3 (100%)	-	8 (100%)	-	7 (100%)	-	4 (80%)	1 (20%)	5 (100%)	-	2 (100%)	-
MEM	12 (92.3%)	1 (7.7%)	3 (100%)	-	8 (100%)	-	7 (100%)	-	4 (80%)	1 (20%)	5 (100%)	-	2 (100%)	-
LEV	12 (92.3%)	1 (7.7%)	-	-	8 (100%)	-	7 (100%)	-	5 (100%)	-	5 (100%)	-	2 (100%)	-
CIP	12 (92.3%)	1 (7.7%)	3 (100%)	-	8 (100%)	-	7 (100%)	-	5 (100%)	-	5 (100%)	-	2 (100%)	-
A/S	10 (76.9%)	3 (23.1%)	3 (100%)	-	5 (62.5%)	3 (37.5%)	7 (100%)	-	1 (20%)	4 (80%)	4 (80%)	1 (20%)	2 (100%)	-
AK	4 (30.8%)	9 (69.2%)	2 (66.7%)	1 (33.3%)	2 (25%)	6 (75%)	4 (57.1%)	3 (42.9%)	2 (40%)	3 (60%)	3 (60%)	2 (40%)	-	2 (100%)
AMC	3 (23.1%)	10 (76.9%)	2 (66.7%)	1 (33.3%)	-	8 (100%)	2 (28.6%)	5 (71.4%)	-	5 (100%)	-	5 (100%)	-	2 (100%)
AM	3 (23.1%)	10 (76.9%)	1 (33.3%)	2 (66.7%)	-	8 (100%)	1 (14.3%)	6 (85.7%)	-	5 (100%)	-	5 (100%)	-	2 (100%)
GEN	4 (30.8%)	9 (69.2%)	2 (66.7%)	1 (33.3%)	1 (12.5%)	7 (87.5%)	4 (57.1%)	3 (42.9%)	2 (40%)	3 (60%)	1 (20%)	4 (80%)	2 (100%)	
P	3 (23.1%)	10 (76.9%)	2 (66.7%)	1 (33.3%)	-	8 (100%)	2 (28.6%)	5 (71.4%)	-	5 (100%)	-	5 (100%)	-	2 (100%)
L	3 (23.1%)	10 (76.9%)	1 (33.3%)	2 (66.7%)	-	8 (100%)	1 (14.3%)	6 (85.7%)	-	5 (100%)	-	5 (100%)	2 (100%)	
K	3 (23.1%)	10 (76.9%)	2 (66.7%)	1 (33.3%)	-	8 (100%)	2 (28.6%)	5 (71.4%)	-	5 (100%)	-	5 (100%)	-	2 (100%)

*IPM* Imipenem, *MEM* Meropenem, *LEV* Levofloxacin, *CIP* Ciprofloxacin, *A /S* Ampicillin-sulbactam, *AK* Amikacin, *AMC* Amoxicillin-clavulanic acid, *AM* Amoxicillin, *GEN* Gentamicin, *P* Penicillin, *L* Lincomycin, and *K* Kanamycin

*α-*hemolytic *S. equi: α-*hemolytic *Streptococcus equi* subspecies *equi.* β-hemolytic *S. equi*: β-hemolytic *Streptococcus equi. K. pneumoniae: Klebsiella pneumoniae. S. aureus: Staphylococcus aureus. P. aeruginosa: Pseudomonas aeruginosa. E. coli: Escherichia coli.* CNS: coagulase negative staphylococci. S: sensitive. R: resistant

### Outcomes of treatment

Out of 36 treated foals, 33 foals (91.7%) with GP tympany and empyema recovered after 4–6 weeks of treatment. All treated foals were followed out for six months and no recurrence was detected in 33 recovered foals. Three cases/36 (8.3%) showed recurrence of GP tympany after two weeks of removal of catheters. The recurrent cases were subjected to laser fenestration of both GPs into pharyngeal cavity. The overall mortality rate was 5.3% (2/38 cases) in foals with GP tympany and empyema. The mortality rates were 0% (0/25 cases) and 15.4% (2/13 cases) in foals with GP tympany and empyema, respectively.

## Discussion

Arabian horse is one of the oldest domesticated horse breeds in the world, it is valued for its grace, beauty, and long-lasting athleticism. As a breed, it is susceptible to GP disorders which affect the animal performance and lead to respiratory disorders [29–32]. Therefore, this study puts in records the data of 38 foals with GP tympany or empyema and the outcomes of their treatment.

In the current study, GP tympany and/or empyema were the commonly recorded GP disorders in the Arabian foals. This is in agreement with previous results [33]. Neonatal foals, especially those in their first month of life, have experienced GP tympany, which can be unilateral or bilateral [5, 9]. In contrast to the findings of a previous study [9], bilateral GP tympany was more common in the current investigation than unilateral. This discrepancy may be caused by a genetically connected sickness unique to the breed of Arabian foals. GP tympany was more frequently observed in females than in males. This result is consistent with earlier research [5, 8]. For unclear causes, fillies are frequently impacted [5].

Although the exact cause of GP tympany is unknown, suggestions include pharyngeal musculature dysfunction or mucosal fold dysfunction [10]. Therefore, it will be interesting to search about the embryological development of the mucosal flap of the GP opening in the future. In addition to the genetic susceptibility caused by the narrow genetic pole in Arabian horses, the severe head curvature of the afflicted Arabian foals may also be a contributing factor in the current study. This suspected factor was mentioned in earlier studies but it needs further research [10, 20].

Clinical, radiographic, and endoscopic investigations are used to diagnose GP tympany [5]. Ultrasonography was not very useful in the current investigation for diagnosing GP tympany due to presence of air inside the GP. Similar findings were previously reported [5, 34]. Ultrasonographic examination can eventually be used to identify fluid within the GP or to evaluate the soft tissues located ventral and lateral to the GP, despite the fact that the presence of air in the GP restricts its efficacy [32]. Since endoscopy allows for a direct view of the GP, it is considered the gold standard for its evaluation [32]. In this study, endoscopy revealed characteristic findings on all examined foals. This is in agreement with an earlier study [32]. Radiography is usually used as the first diagnostic imaging to support endoscopic findings, despite its limitations due to superimposition [5, 32]. Cross-sectional imaging, especially computed tomography (CT), becomes more widely available. CT appears to be the best way to fully assess the surrounding soft tissues and precisely identify lesions of the tympanostylohyoideum, temporal bone, and skull base that are affected with GP disease [28, 32].

There are no hematologic abnormalities that are pathognomonic for GP tympany or empyema in foals. Foals with uncomplicated GP tympany often have normal hematology and serum biochemistry because the condition is primarily a mechanical obstruction rather than an inflammatory disease. A recent study did not identify a characteristic CBC profile in Arabian foals with GP empyema [35]. Although hematologic abnormalities are generally nonspecific, foals with GP empyema exhibited inflammatory leukograms characterized by neutrophilia and hyperfibrinogenemia and mild hyperproteinemia due to inflammation and dehydration. These findings are similar to those reported before [5]. Therefore, CBC and serum biochemistry are supportive rather than diagnostic for GP disease. Endoscopy and bacterial culture remain the diagnostic gold standard [35].

Excessive purulent nasal discharge is a typical clinical sign of GP empyema that is unrelated to activity. Additionally, it was noted as bilateral and unilateral. GP empyema was more prevalent in females than in males. These results are consistent with earlier research [5, 8]. When the GP has exudations, ultrasonography helps for diagnosing of some cases of GP empyema. An earlier study revealed similar findings [20]. In the current investigation, ultrasonography revealed hypoechoic fluid in cases of bacterial infection and hypoechoic fluid mixed with hyperechoic particles in cases of mixed bacterial and fungal GP infections. In this work, endoscopy revealed very characteristic findings in foals with GP empyema which help in verifying diagnosis and assessment of the severity of the disease. These findings are consistent with results recorded before [32].

In the present study, samples were taken from all cases of GP tympany and empyema and showed double and triple mixed infections. Therefore, a possibility of infectious cause of GP tympany is suggested, in addition to the other supposed causes. Thus, it is recommended to perform culture and sensitivity test in all cases of GP tympany and/or empyema in foals for proper diagnosis and successful treatment. Similar results were reported by van der Vossen et al., who noted that no pure culture of a single microbe had been detected in any of the foals with GP empyema [35].

In the current study, *Streptococcus equi* ssp. *equi* is the most frequently isolated bacteria from foals with GP empyema and complicated tympany. Therefore, in all cases with GP empyema and complicated GP tympany, isolation protocols should be followed until culture results are available due to the high contagiousness of *S. equi* ssp. *equi* infection (strangles). This is in agreement with the results of previous studies [5, 35–37]. Nevertheless, other bacteria could be isolated from the examined foals. Gram-negative bacteria such as *Klebsiella pneumoniae*, *Pseudomonas aeruginosa*, and *Escherichia coli* have been increasingly documented in respiratory swabs of affected horses, typically complicating primary streptococcal infections [37].

Regarding treatment of foals with GP tympany and empyema, the outcome was excellent (91.7%) and the recurrence rate was 8.3% only. This could be attributed to the early diagnosis and prompt treatment with the most suitable antibiotics according to the results of the sensitivity test.

Antibiotic susceptibility data include many compounds that are either on the critical list of antibiotics or, depending on the country for reservation for human usage. These susceptibility results indicate that the isolated bacteria are multi-drug resistant (MDR) to traditional first-line veterinary antibiotics (Penicillin, Lincomycin, Kanamycin), but highly susceptible to potent broad-spectrum and advanced-generation antimicrobial classes (Carbapenems, Fluoroquinolones). For these particular MDR isolates, first-line therapies like Penicillin, which are commonly used for respiratory pathogens like *Streptococcus equi* spp *equi*, are ineffective. Similar findings were mentioned in a recent study [38]. Despite their great efficacy, fluoroquinolones and carbapenems are often saved for serious, life-threatening infections in order to stop the spread of microbial resistance. Fluoroquinolones should be administered cautiously in foals because of the possibility of joint cartilage toxicity [39].

In this study, GP tympany and empyema were treated by endoscopic placement of a Foley catheter and left in place for 4–6 weeks with frequent lavage with normal saline and infusion of appropriate antibiotics according to the results of the sensitivity test. The success rate was 91.7% in foals with GP tympany and empyema. In this regard, 80% of Foley catheter placements were successful in foals [19].

Good decompression of the GP is the aim of treatment for GP tympany [5]. In the current investigation, we were able to effectively decompress and drain the contents of the GP by placing a transnasal Foley catheter and flushing it for four to six weeks. A similar method has been used with positive results in foals with GP tympany [40].

Good drainage of the GP contents is the aim of treatment of GP empyema [5]. Fenestration of the septum between the GPs, either with or without ipsilateral pharyngeal opening extension or mucosal fold resection, is one of the techniques suggested for achieving this goal [41]. Making a fistula with the throat has been described as another method of achieving drainage. For drainage, Viborg’s triangle approach is also employed. Grasping the median septum creates an opening that is two to three centimeters in diameter. Alternatively, an endoscope is placed into the normal side and the septum excision is performed under endoscopic supervision. Because the septum extends into the unaffected side, it is simple to grasp the mucosa. It is essential to make an incision that is at least 2 cm in diameter because sealing can result in recurrence. Furthermore, a transendoscopic laser fenestration technique has been documented [42]. In order to effectively drain the contents of the GP and flush with the appropriate antibiotics for four to six weeks in addition to systemic antibiotics, we used a transnasal Foley catheter in this trial. To prevent infections from spreading to the lower respiratory tract and local lymph nodes, systemic antibiotics were also administered. Similar results have previously been reported [5].

In our work, we employed a transendoscopic laser fenestration technique for effective drainage in three recurrent cases. Chronic GP empyema may cause scarring of the pharyngeal entry, which may prevent the affected side from draining [5]. Fenestration by itself works well if the problem is unilateral. However, if bilateral involvement is discovered, pharyngeal opening extension is also required. This can be accomplished by grasping and resecting the mucosal fold [5].

This retrospective investigation has many limitations, such as being restricted to a single center and having low number of cases as well as short follow-up time to ascertain the real prognosis.

For microbiological sampling of foals with GP empyema and complicated tympany using an endoscope and a sterile Foley catheter, the main limitation of the sample collection and culture procedures is the potential contamination from the upper respiratory tract. Despite careful sterilized technique, transient contamination cannot be completely excluded. Moreover, potential contamination from the endoscope even with appropriate reprocessing remains a theoretical limitation of endoscopic sampling. The Foley catheter may collect material from only one area of the GP and bacteria may not be uniformly distributed within the GP, particularly when thick pus or chondroids are present which is considered a sampling bias.

Culture-dependent limitations include: delays between collection and laboratory inoculation may reduce recovery of fragile organisms, limited detection of anaerobes, and single-time-point sampling. Additionally, conventional aerobic culture detects only viable, cultivable bacteria, while fastidious, anaerobic, or non-cultivable organisms may be missed. Also, mixed infections may be incompletely characterized if one organism overgrows others,

## Conclusions

In the light of this study, GP tympany and/or empyema commonly affect young purebred Arabian foals, particularly females. Foals with bilateral GP tympany and/or empyema are higher than foals with unilateral disorder. Arabian foals with GP tympany and/or empyema have characteristic clinical signs and endoscopic findings and nonspecific findings of hematology and serum chemistry. Although ultrasonography is a helpful diagnostic technique for foals with GP empyema and complicated GP tympany, endoscopy is the gold standard for verifying the diagnosis and planning the treatment of GP illness. All foals with empyema and complicated GP tympany should be subjected to culture and sensitivity test because some cases of GP tympany may have an infection. Foals with empyema and complicated GP tympany have double and triple mixed bacterial and fungal infections. *S. equi* ssp. *equi* is the commonly isolated bacteria from these foals. GP tympany and/or empyema can be treated successfully by placement of a transnasal Foley catheter under the guidance of endoscopy with frequent lavage and infusion of suitable antibiotics. Recurrent cases require transendoscopic laser fenestration.

## Data Availability

All relevant data supporting the findings of this study are included within the manuscript. The raw datasets used and/or analysed during the current study are available from the corresponding author on reasonable request.
